# Exploring adults’ recollections of growing up with childhood motor difficulties: a qualitative study using systematic text condensation

**DOI:** 10.1136/bmjopen-2024-084346

**Published:** 2024-08-08

**Authors:** Johanna Zahlander, Anna Fäldt, Amanda Kirby, Kine Johansen

**Affiliations:** 1 Department of Women’s and Children’s Health, Uppsala University, Uppsala, Sweden; 2 Department of Public Health and Caring Sciences, Uppsala University, Uppsala, Sweden; 3 University of South Wales, Pontypridd, UK

**Keywords:** developmental neurology & neurodisability, neonatology, quality of life

## Abstract

**Abstract:**

**Objective:**

To explore the ramifications of childhood motor difficulties, providing insights into their impact and consequences over time.

**Design:**

A qualitative study using semistructured individual interviews. Data were analysed using systematic text condensation.

**Setting:**

Neonatal intensive care recipients born at Uppsala University Children’s Hospital, Uppsala, Sweden, between 1986 and 1989, were enrolled in a longitudinal follow-up study and subsequently interviewed in 2019–2020.

**Participants:**

13 individuals in their early 30s, who met the criteria for developmental coordination disorder or performed below the 5th percentile on motor tests at 6.5 years of age, were interviewed. Those with co-occurring deficits in attention or social behavioural at age 6.5 were excluded.

**Results:**

Two themes emerged: (1) lifelong challenges and (2) navigating the journey of motor difficulties: support, awareness and confidence. Five participants reported persistent motor difficulties. They adapted and integrated these challenges into their daily lives without feeling constrained. Parental support was crucial to their success, whereas support from schools was limited.

**Conclusion:**

Adults who faced motor difficulties in childhood developed effective coping strategies, overcame challenges and now lead fulfilling lives. The findings stress the importance of parental support and understanding, addressing contextual factors and fostering positive attitudes and supportive environments to enhance well-being and participation.

**STRENGTHS AND LIMITATIONS OF THIS STUDY**

- This study fills a recognised knowledge gap by exploring the experiences of individuals with motor difficulties, from childhood to adulthood.
- The absence of reassessment in adulthood hinders the objective evaluation of motor difficulties and confirmation of a diagnosis of developmental coordination disorder.
- The option of face-to-face or phone interviews could enhance participation by overcoming practical barriers and reaching and including many eligible participants. However, different interview modes might impact the information shared.
- Acknowledging potential unawareness of prior involvement in research, participants were given the opportunity to inquire about childhood assessments and motor difficulties. They willingly shared their experiences.

## Introduction

 Developmental coordination disorder (DCD) is a neurodevelopmental disorder that adversely affects the ability to learn and perform motor skills at an age-appropriate level, significantly interfering with daily activities including academics, leisure and play.^1^ This common motor disorder, affecting approximately 5%–6% of children,^1 2^ emerges early in life and is not attributable to intellectual disability, visual impairment or neurological conditions affecting movement.^1^ Children with DCD tend to participate less in physical and social activities than their peers,^3 4^ potentially impacting both physical and mental health.^2 5 6^ Additionally, DCD frequently co-occurs with other neurodevelopmental disorders such as attention deficit hyperactivity disorder and autism spectrum disorder.^2 7^

Growing evidence suggests that DCD is not merely a childhood condition but a lifelong disorder,^2 8^ with motor difficulties persisting throughout life and presenting new challenges in various life phases.^2 8 9^ Difficulties in executive functions, commonly observed in children with DCD, tend to either increase or become more apparent over time.^2^ Activities requiring organisational skills, such as planning the day or preparing meals, pose significant challenges.^2 9^ As individuals age, there is an increasing demand for organisational skills,^9^ and difficulties in handwriting, planning and organising daily tasks can significantly affect the ability to finish higher education and maintain gainful employment.^810^,^12^ Although the research is scarce, studies indicate that those diagnosed with DCD in childhood tend to spend fewer hours per week being physically active and exhibit more sedentary behaviour compared with their peers.^13 14^ Furthermore, studies involving adolescents, young adults and adults with DCD have shown that persistent functional impairments are linked to reduced quality of life, diminished life satisfaction and limited participation in various settings such as avoiding team sports and choosing not to attend clubs or go dancing.^8^,^10^

The increasing focus on DCD beyond childhood has provided valuable insights into its effects on daily life and the long-term psychological and social consequences.^8^,^1015^ For effective intervention and treatment planning, an understanding of how individuals manage their motor difficulties over time is required.^15^ Effective coping strategies can help with symptom management while dysfunctional coping might perpetuate symptom maintenance.^15^ Among individuals with DCD, withdrawal and avoidance are frequently reported as common maladaptive coping strategies.^815^,^17^ Conversely, common adaptive coping strategies used by adults with DCD include employing cognitive strategies, such as spending more time on tasks and making environmental modifications.^15^ Despite facing numerous constraints from their motor difficulties,^15 18 19^ individuals with DCD might compensate for their symptoms to the extent that they are not overtly observable.^15 18^ Additionally, while motor issues can cause frustration and stigma throughout life, the psychosocial and psychiatric challenges associated with DCD often have a more significant impact.^6^

Although there is growing awareness of the persistent nature of DCD, there is a scarcity of longitudinal studies tracking its trajectory into adulthood.^2^ Without an understanding of developmental trajectories and commonly employed coping strategies, interventions cannot be optimised. Information about predictors of persistence and protective factors is crucial to prevent secondary consequences and promote both physical and mental health. Therefore, this study aimed to explore the impact of growing up with motor difficulties, providing insights into the impact and implications of these challenges over time.

## Material and methods

### Study design and participants

In this qualitative study, we conducted semistructured individual interviews with participants recruited from a cohort of neonatal intensive care (NIC) recipients born at Uppsala University Children’s Hospital, Sweden, between 1986 and 1989. The initial longitudinal follow-up study included all surviving infants (n=226) who received treatment at the NIC unit and were residents of Uppsala County.^20^ At the age of 6.5, their motor abilities were assessed using the Test of Motor Impairment (TOMI), Henderson revision,^21^ the predecessor of the Movement Assessment Battery for Children (MABC),^22^ and the Motor-Perceptual Development (MPU), 0–7 years.^23^ Based on this assessment, Hemgren and Persson retrospectively categorised the children based on their motor abilities into different groups: those with no motor deviations, motor delay, moderate and definite motor problem, and moderate and definite DCD.^24^ These categories were defined using TOMI, MPU, and the Diagnostic and Statistical Manual of Mental Disorders Text Revision Fourth Edition criteria for DCD.^1^ The severity of the motor problem was determined using the percentile cut-offs on TOMI.^21^ Falling between the 6th and 15th percentiles indicated a moderate motor problem or DCD while performing below the 5th percentile was defined as a definite motor problem or DCD. Since this classification was done retrospectively, participants were not informed about their specific motor deviation categories. Nevertheless, they were offered physiotherapy as needed, following regular practice.

In the present study, we invited individuals who, at 6.5 years of age, fulfilled the diagnostic criteria for DCD or performed below the fifth percentile on TOMI, residing in Sweden in 2019. To better understand how motor difficulties affected individuals and to minimise the potential confounding effects of co-occurring disorders, we excluded participants with an attention deficit or a deficit in social behaviour according to the Combined Assessment of Motor Performance and Behaviour at the time of assessment.^25^ At the initial assessment at 6.5 years of age, children with congenital malformations, cerebral palsy, impaired hearing, mental retardation or Tourette syndrome were excluded, and therefore, not eligible for this study.^24^ A total of 25 individuals met the inclusion criteria, out of which 18 were contacted. We used a convenience sample, initially inviting those residing in or near Uppsala County (n=14, women n=4), and later extending the invitation to participants throughout Sweden. To ensure gender balance, we adopted a strategic selection process, involving drawing lots with a 2:1 ratio (men:women). This selection process was a collaborative effort between the first author (JZ) and the last author (KJ).

### Data collection

At the end of 2019, a study invitation was sent by post. Approximately 3 weeks later, the first author (JZ) phoned potential participants to inform them about the study and their previous participation in the initial longitudinal follow-up study. Two potential participants did not respond to the call, and one declined to participate without providing any specific reason. During these conversations, she introduced herself by name and profession, explained the purpose of the call and provided participants with the opportunity to pose questions about the study. Since parental consent was obtained in the initial longitudinal follow-up study, some participants might have been unaware of their prior involvement in research and the outcomes of their childhood assessments. This may have led to participants learning about their childhood motor difficulties for the first time on receiving information about the study, potentially causing discomfort. To address this concern, participants were given the opportunity to inquire about their childhood assessments and motor difficulties during both the initial phone call and the subsequent interview.

All interviews were scheduled based on the participants’ preferences, either as a face-to-face interview (n=6) or a telephone interview (n=8). For face-to-face interviews, participants preferred the setting to be within the university’s premises, conducted in a private space. In the case of telephone interviews, participants were asked to choose a convenient time and place where they could talk without being undisturbed. All interviews were conducted in January and February 2020. Written or verbal consent was obtained before the interviews.

The interviews followed a semistructured interview guide based on a previous study by Zwicker *et al*,^26^ which was further modified by the authors to better suit adult participants (online supplemental file 1). All interviews were conducted by a female physiotherapist (author JZ) without prior contact or knowledge of the respondents. To ensure accuracy, the interviewer summarised the conversation at the end of each interview, allowing participants the opportunity to correct or add information. Further opportunities to make corrections to the transcripts were not given.

After the fourth interview, questions about childhood physical activities and support systems were added to the interview guide. Subsequently, previously interviewed participants were contacted by phone to address these additional questions. All interviews were audio recorded and transcribed verbatim by the first author (JZ). The interviews, on average, lasted 42 min (IQR 25–49.5 min). After the fourteenth interview, our data included diverse descriptions of participants’ experiences growing up and no new information emerged, that is, saturation was reached and data collection was ended.

Additionally, 12 participants completed a questionnaire aimed at capturing background data.

### Data analysis

The data underwent an inductive analysis using systematic text condensation (STC), a descriptive and explorative method that offers a pragmatic approach to thematic cross-case analysis of qualitative data.^27^ The STC procedure comprises four steps (table 1). Initially, all transcripts were read thoroughly to gain a total impression of the content. The first and last authors (JZ and KJ, respectively) identified preliminary themes and engaged in subsequent discussions to reach an agreement before categorising these themes into code groups. The second author (AF) reviewed the code groups and provided feedback. Next, the content within each code group was decontextualised, systematically abstracted and rephrased into a narrative attributed to a single person. Finally, these condensed narratives were described and conceptualised.

**Table 1:** An example of the analysis procedure according to systematic text condensation

Steps in the analysis process		Examples from the text
1	Total impression	The participants described several different coping strategies they used in daily life.
	Identify themes	Preliminary theme: Coping strategies
2	Identifying and sorting meaning units	Text: *‘*You can’t give up. You try again and again. But then you may have to give up and say, ‘enough is enough’
	From themes to codes	Code group: Coping strategies for mastering motor skills
3	Condense the meaning units as if it was narrated by one participant	Condensation: ‘It is important to persevere, try again and again but sometimes it is okay to give up. I can take the escalators instead of the stairs or I can sit down when putting on my pants. Sometimes you might choose to focus on one aspect of a task; for example, concentration on leg movements in a gym class choreography, once comfortable, adding the arm movements.
	From code to meaning	Summary: Coping strategies were needed in certain daily activities.
4	The condensations are synthesised, described and conceptualised.	Essence: The participants had developed their own strategies to cope with their motor difficulties, and they felt confident using them whenever needed.Category: From challenges to confidence
	From condensation to descriptions and concepts	When reading through the interviews as a whole, we experienced that the themes and categories were consistent with the statements in the interviews.

The Consolidated Criteria for Qualitative Studies (COREQ) was used to ensure that essential information regarding the study was reported.^28^

### Patient and public involvement

Patients or members of the public were not involved in this study.

### Reflexivity

The research group comprised two female physiotherapists (JZ and KJ) specialising in preschool children with diverse motor difficulties, a female speech and language therapist (AF) with expertise in working with children with disabilities, and a female medical doctor (AK) with over two decades of clinical and research experience focused on children and adults with DCD (AK). Two of the authors (KJ and AF) have extensive experience in qualitative research methodologies.

To enhance the confirmability of the analysis and mitigate the impact of research bias,^29^ the analytical process was conducted independently by two researchers (JZ and KJ). Thereafter, a third researcher (AF), without extensive knowledge about motor difficulties in children, reviewed the themes and condensations. The findings were discussed until a consensus was reached regarding the analysis and interpretation of the individual themes.

## Results

### Participants

Interviews were conducted with 14 individuals, 4 of whom were women (table 2). One participant was excluded from the analysis after her interview revealed a childhood epilepsy diagnosis. The participants’ ages ranged from 31 to 34 years (median: 32.0 years, IQR: 31.5–33.0), and eight were born preterm (median: 35 gestational weeks, IQR: 31.0–40.0). Among them, 10 participants were employed, 1 was on parental leave and another attended university. Six participants had completed high school or an equivalent of 12 years of education, three had attended university or college but not completed their degree and three were postgraduates. Six participants had partners and four of whom had children. Sociodemographic data for a male participant were missing.

**Table 2:** Participant characteristics

	TotalN=13 (100)	Persistent motor difficultiesN=5 (38.5)
Gestational age		
28–31	4 (31)	1 (20)
32–36	4 (31)	1 (20)
Full term	5 (38)	3 (60)
Sex		
Female	3 (24)	2 (40)
Male	10 (76)	3 (60)
Motor ability at 6.5 years		
DCD	9 (69)	4 (80)
˂5th percentile, TOMI	4 (31)	1 (20)
Highest education level		
Completed high school or 12 years of schooling	6 (45)	1 (20)
Education at least 1 year after high school	4 (31)	3 (60)
Degree from university or college	2 (16)	1 (20)
Missing data	1 (8)	
Current status		
Student	1 (8)	1 (20)
Employed	10 (76)	4 (80)
Parental leave	1 (8)	
Missing data	1 (8)	

DCDdevelopmental coordination disorderTOMITest of Motor Impairment, Henderson revision

Prior to our study, one participant had been diagnosed with dyslexia, while another reported having asthma and allergies. None of the other participants reported any neurodevelopmental disorders or chronic conditions.

The interviews revealed that five participants (38.5%) experienced persistent motor difficulties; of these, four were diagnosed with DCD at 6.5 years of age, three were born full term and two were female.

### Analysis of qualitative data

Two themes emerged during the analysis process: (1) lifelong challenges and (2) navigating the journey of motor difficulties: support, awareness and confidence (table 3). The themes consisted of two and three categories, respectively, presented below with illustrative quotes. Pseudonyms are used in the presentation of findings to protect the privacy and identities of the participants.

**Table 3:** Overview themes and categories

Themes	Categories
Lifelong challenges	Recalling the journey: navigating life with motor difficulties from childhood to adulthood. The journey beyond motor difficulties: emotional and cognitive challenges across the lifespan.
Navigating the journey of motor difficulties: support, awareness and confidence	It would not have been possible without them: parental support and overcoming motor difficulties. Self-perception and motor skills: a journey of awareness. From challenges to confidence.

#### Theme 1: lifelong challenges

##### Recalling the journey: navigating life with motor difficulties from childhood to adulthood

While many participants expressed facing challenges in performing and participating in various activities during childhood and adolescence, such as playing with others, engaging in physical activities and participating in sports, over half of them did not recall experiencing specific motor difficulties during that time. Instead, they attributed these challenges to factors such as a perceived lack of talent for a particular sport, difficulties in maintaining concentration during activities or physical aspects such as being overweight or having asthma.

The fact that I was not the best at sports in the class was probably not because I was retarded. It was because the level of my classmates was high.—Daniel

As adults, some participants described experiencing coordination issues, such as difficulties in adjusting the force or pace of their movements.

I tend to stumble too. […] It can happen at any time, on a carpet edge or on a stone in the street. It has been like that as long as I can remember.—Emelie

One participant mentioned having difficulty in learning how to ride a motorcycle due to balancing issues while others shared stories of struggling to learn how to drive. Some had developed exaggerated adverse reactions to certain activities, such as exercising in a gym with others.

I have huge difficulties in situations where you have to have certain intricate control over your body. Especially in situations that include several body parts simultaneously, such as intimate and sexual situations and… And, when you’re performing more advanced exercises. —Linus

Activities requiring bilateral coordination and balance were challenging for some participants, while others reported clumsiness and difficulties manipulating small objects.

I usually say that I can manage everything well until it reaches the wrist, then it stops.—Michael

None of the participants who were parents reported that their motor difficulties, whether persistent or earlier, affected their abilities as parents.

### The journey beyond motor difficulties: emotional and cognitive challenges across the lifespan

Half of the participants, including both those experiencing persistent motor difficulties and those without, expressed facing challenges extending beyond motor difficulties. This involved coping with experiences of depression or difficulties in controlling their temper, resulting in anger outbursts, destructive behaviours or physical altercations. Although participants noted that temper control tended to improve with age, it remained a persistent and troublesome concern for many.

Well, it’s probably irritation and stuff like that, in the brain. It explodes, so to speak.—Michael

During childhood, some participants recalled struggling with learning and classroom activities.

I was always the one who couldn’t sit still. I guess I wasn’t the most enjoyable pupil to have. […] It was very difficult for me to concentrate on what I was supposed to do instead of everything else happening around me.—Victor

As adults, some mentioned being easily distracted at work. Several emphasised the importance of planning their day to maintain a sense of control and avoid mistakes. In contrast, others described difficulties in making plans, following through with them, or postponing activities until it was too late.

I had to call the company and ask them to postpone the invoice. Because I had wasted the money on other things.—Andreas

Some participants attributed these challenges to a lack of discipline. They explained that difficulties in planning had a negative impact on their academic performance during childhood and adolescence and continued to affect their work performance and relationships with friends or family in adulthood.

Experiences of bullying and feeling excluded during childhood and adolescence were shared by some participants, who sometimes directly attributed these negative experiences to their motor difficulties.

### Theme 2: navigating the journey of motor difficulties: support, awareness and confidence

#### ‘It would not have been possible without them’: parental support and overcoming motor difficulties

The participants recounted substantial parental support throughout their childhood and adolescence, which extended to various daily tasks, managing school work and participating in recreational activities. This encompassed learning tasks such as tying shoelaces, riding a bike, swimming, reading and assistance with homework. Additionally, parents provided invaluable emotional support and encouraged their children to explore new activities despite their motor difficulties. Many attributed their ability to overcome childhood motor difficulties to the relentless efforts of their parents.

I guess it’s because my parents pushed me. Do this. Try this. So, it was surely thanks to them.—Oscar

Several participants shared experiences of lacking social support at school, encountering confrontations with teachers and feelings of exclusion. With the exception of one participant who received extra physical education lessons, none of the participants could recall receiving additional support for their motor difficulties at school.

Being physically active was described as a natural part of upbringing, and participating in sports activities was portrayed as the norm. Male participants described active engagement in team sports from a young age until their late teens, feeling included and encouraged to participate in recreational activities regardless of difficulties.

I played football when I was a kid. It was in the countryside, so everyone was allowed to join; it wasn't like an elite programme or anything like that. Even if you weren’t very good, you were allowed to participate, so to speak.—Michael

In contrast, some women mentioned a lack of support, and none had participated in team sports; instead, they engaged in individual sports or other activities during their leisure time.

Although parental support was mainly mentioned when the participants talked about their childhood, some described a continuous need for support as adults.

Now I have a wife who helps me quite a lot with planning, but, of course, many of our disputes start there.—Eric

One participant expressed a desire to alleviate the emotional burden on his parents by demonstrating that his motor difficulties did not cause significant suffering. He also wished for his parents to receive support in coping with the impact of his motor difficulties.

#### Self-perception and motor skills: a journey of awareness

Participants expressed feelings of being different from others and described themselves as clumsy, big and awkward. Many regarded motor difficulties as an inherent personal trait rather than actual motor problems. Some participants mentioned that they only became aware of their motor difficulties when someone pointed it out or as they grew older and noticed a recurring pattern.

My husband says I’m a bit clumsy, but it’s not something I struggle with.—Emily

A few participants stated that participating in the study and realising that their motor difficulties could explain certain behaviours or traits had the potential to alter their self-perception and the way they viewed their difficulties.

I don’t think I have reflected upon having motor difficulties. It just is. It’s interesting to think about it from that perspective. I’ve just thought about it like “This is me, my personality, being silly and tripping and clumsy and so”… So maybe, it might be a bit easier if it gets a… It might be that it’s because I have motor difficulties… that makes me act as I do. Maybe it will be easier to understand.—Anna

Others described how their awareness of having motor difficulties influenced their self-efficacy and made them more self-conscious.

I may have questioned myself a bit more because I got that label of having poor motor skills. Perhaps it’s not the actual motor skills that have hindered me, but rather the mental ones. When someone has said, “This is you”, instead of listening more to myself and thinking, “No, but this is me”.—Christopher

Transitioning into adolescence, their activities shifted towards more social interactions, such as spending time and talking with friends. Many found this phase of life more accessible and felt that these non-physical activities suited them better. The transition to adulthood involved a positive re-evaluation of personal traits within a social context, where social activities gained prominence over physical ones. However, those not recalling motor difficulties often did so without attaching specific emotional context. Being good at something and being able to perform well, usually in areas other than sports, played a role in how they viewed themselves. Overall, the transition into adulthood was generally described positively by the participants.

I'm clumsy; it’s not the end of the world.—Simon

### From challenges to confidence

Participants shared various strategies and adaptations employed throughout their upbringing and as adults, depending on the specific situation, task and motivation. A common approach was to assess their performance beforehand by evaluating whether they had the necessary skills or by stepping back and re-evaluating the situation when they encountered difficulties. Many participants adopted a strategy that involved taking their time and finding their own way to complete the task. They also emphasised the importance of persistent practice in overcoming their challenges. Several participants who did not report ongoing difficulties attributed their success in motor activities to their perseverance, considering this a key trait in their achievements. Similarly, participants facing ongoing challenges also found that practice often paid off.

It worked out when I found my technique. I'm the kind of person that if I decide this is what I'm going to do, then I can sit and practice it, frantically.—Christopher

Nevertheless, they acknowledged that some tasks might be abandoned after repeated failures.

You can’t give up. You try again and again. But then you may have to give up and say, “enough is enough”.—Sarah

They also stressed the importance of not letting pride or shyness hinder them from seeking help when needed. Some participants emphasised the importance of their own reactions and self-perception in managing and handling their difficulties when facing failure.

A common strategy involved avoiding certain activities, such as opting for the escalator instead of stairs, having drinks before a job meeting to prevent spills or refraining from specific activities.

I guess I always have a hard time exposing myself to new situations, if someone wants to try an activity I’ve never tried before. Something that I’m not sure I can master; it could be like a picnic. Like how to get there, what to bring, practical things. That has to do with motor skills. Clumsiness, spilling things […] It makes me avoid things like that. I avoid new things….—Linus

Regardless, none of the participants felt limited in their daily lives. Instead, they expressed confidence in their bodies and their ability to lead the lives they desired, capable of performing desired activities, whether they used strategies or adaptations for success or not. With age, they had learnt to live with their motor difficulties and had gained self-confidence, even as the challenges of motor difficulties persisted. ‘I don’t think there is anything I can’t do’.—Anna

## Discussion

This study explored the experiences of adults who had motor difficulties as children, examining the impact of these challenges on their daily lives, both in the past and the present. Over time, participants developed strategies to navigate these challenges, with none feeling limited in their adult lives. Those with persistent motor difficulties reported issues with coordination, balance and manipulating small objects, often describing themselves as clumsy and awkward. Despite fewer than half of the participants reporting persistent motor difficulties, many still faced challenges in various activities, often attributing them to factors beyond motor skills. All participants expressed confidence in their bodies and their ability to lead a life on their terms, employing specific strategies, making adjustments or using adaptations to participate fully.

It is well established that preterm infants follow a different motor development trajectory compared with full-term infants.^30^ However, the distinction between preterm and term DCD is not always clear,^31^ with literature suggesting that the severity of motor difficulties^32 33^ and distinct cognitive and motor profiles influence outcomes.^34 35^ Furthermore, one study showed that while children born preterm exhibit improved motor function over time,^31^ those born at term tend to display consistently low and stable rates of DCD. Notably, in our study, a higher percentage of participants diagnosed with DCD at 6.5 years who were born at term continued to experience motor difficulties into adulthood.

Our study also revealed noticeable gender differences regarding participation in team sports. While all men had actively participated in team sports during their upbringing, expressing a sense of inclusion and encouragement, women reported a lack of support and had not participated in team sports. These findings align with research involving young adults with motor difficulties,^16^ highlighting gender differences in expectations for sports performance. This difference might be attributed to culturally and socially constructed beliefs assuming inherent differences in motor abilities between boys and girls,^36^ potentially leading to divergent exposure to physical activities during childhood and adolescence, resulting in fewer opportunities to practise motor skills.^37^

Similar to research on children and young people with DCD,^11 17 38^ participants in our study often used negative terms when describing themselves. Notably, these negative self-perceptions were rarely attributed to their motor difficulties, but were more frequently perceived as inherent personal traits. Our findings are consistent with those of Missiuna *et al*,^16^ who observed that participants described their clumsiness as a personal characteristic and accepted it as part of themselves. The authors emphasised the significance of the social context for participation when understanding the impact of coordination difficulties on self-image.^16^

In our study, some participants recognised during the interviews that their motor difficulties influenced specific behaviours or traits, potentially altering their self-perception and understanding of the challenges they faced. Previous studies suggest that understanding motor problems and receiving a formal or informal DCD diagnosis can positively impact self-confidence and self-perception.^17 39 40^ Although, the initial longitudinal follow-up study did not formally diagnose participants with DCD, information about the children’s motor abilities was conveyed during follow-up assessments and physiotherapy sessions as needed. While understanding a motor problem or receiving a DCD diagnosis is generally percieved positively, it can also evoke negative or mixed emotions.^40^ One participant expressed that the limiting factor was not poor motor skills themselves, but the label hindering his participation in desired activities. Despite concerns about labelling,^38 41^ research supports the benefits of providing explanations and fostering understanding regarding motor difficulties.

Participants reflected during interviews on how their parents actively supported and encouraged them to try new activities, emphasising the pivotal role of such support in their success. Research highlights the central role of families in fostering children’s skill development and competence,^42^ emphasising the importance of parental support for children’s achievements and well-being.^17^ Recognising that parental behaviours are shaped by their knowledge, beliefs and attitudes underscores the potential impact of enhancing parental awareness and knowledge.^42^ This can serve as a valuable tool to empower families and optimise children’s health and well-being.^6^ When parents and siblings understand the strengths and capabilities of children with DCD, several positive outcomes emerge. These include children experiencing greater ease in navigating daily life, increased engagement within their home and community, and the development of positive self-perceptions.^17^ Our findings indicate that when parents understand their children’s motor difficulties, it can help their children accept and embrace these differences. Additionally, social and attitudinal environments significantly influence acceptance and the opportunity for participation in activities.^17^ One participant highlighted the crucial role his wife played in organising, managing time and handling daily tasks, illustrating the ongoing need to educate close family members or caregivers about the specific needs and support requirements of individuals with DCD throughout their life course.

While our discussion has primarily focused on the influence of parental support and gender in motor development, it is essential to recognise that motor development is a complex biocultural construct shaped by a mix of extrinsic and intrinsic factors.^43^ For example, socioeconomic status influences access to developmental resources such as play materials and enriched environments^44^ while broader social conditions such as family dynamics, cultural norms and educational opportunities shape children’s participation in physical activities.^17^ Intrinsic factors, including genetics, biological maturation and individual neurodevelopmental characteristics, affect the baseline capabilities and the progression of motor skills.^17 43^ Furthermore, medical conditions, including mental health issues, can impact motor competence and physical activity levels.^43^

In exploring the trajectory of motor difficulties from childhood to adulthood, our study found that an improved person-environment fit positively influenced outcomes. This aligns with findings from other studies,^11 16 45^ such as those by Missiuna *et al*, who found that an enhanced fit resulted in improved self-acceptance and a sense of control among individuals with DCD.^16^ This, coupled with increased acceptance by peers, contributed to improved confidence and a more positive self-image. Moreover, individuals with DCD described that their motor skills improved over time or that they learnt to adapt to the demands or context.^16^

To optimise intervention and outcomes, it is therefore essential to understand how individuals with DCD adapt to their daily challenges and the coping strategies they employ. Our results support previous research that shows that in their efforts to cope with the challenges posed by their motor difficulties, individuals with DCD often resort to avoidance or withdrawal strategies.^15^,^17^ Expanding on this theme, a participant in our study shed light on how motor difficulties impacted intimate and sexual relationships, creating a hesitancy to engage in such activities. Alongside avoidance, participants in our study, as well as those documented in the literature,^15^,^17^ employed coping strategies such as perseverance, excelling in specific activities or finding their own way of doing things. Perseverance emerged as a crucial personal trait emphasised by participants for achieving success. This aligns with prior research, suggesting that interests and motivation to engage in activities with peers play a significant role in motivating individuals with DCD to persist and practise challenging tasks.^11 17 38^

Our study suggests that individuals with motor difficulties adeptly navigate their challenges and learn to live with them without feeling limited in everyday life. For those with DCD, learning to accept challenges has proven instrumental in fostering autonomy and confidence in managing various aspects of life.^17^ While research following individuals with DCD over time is scarce, our findings, in line with other studies, indicate that individuals with DCD typically express a positive outlook on their everyday lives in adulthood. They demonstrate a positive sense of self and activity competence, perceiving minimal limitations in their daily life or future aspirations.

Research by Meachon and Alpers suggests that, over time, individuals with DCD develop various strategies to compensate for their motor difficulties, reducing the relevance of physical coping strategies in adulthood.^15^ Instead, cognitive strategies, environmental modifications and social support emerge as crucial elements in interventions.^15^ This is particularly relevant since our participants often faced challenges beyond motor difficulties. With a scarcity of psychologically based therapies and a lack of psychopharmacological interventions for DCD, social support emerges as a potentially beneficial intervention.^6 15^ Looking ahead, a shift from impairment-based approaches to strength-based ecological or environmental assessments and interventions is recommended, aiming to enhance social and community participation for individuals with DCD.^16^

### Strengths and limitations

This article addresses a recognised knowledge gap by exploring the experiences of individuals with motor difficulties, from childhood into adulthood, providing insightful descriptions through the participants’ own narratives. However, it should be noted that the absence of reassessment in adulthood limits our ability to objectively evaluate the persistence of motor difficulties and confirm a DCD diagnosis. This limitation is particularly significant considering that motor difficulties associated with DCD may change or disappear over time.^31^ While our study relied on participants’ recollections, which introduces potential inaccuracies, it primarily aimed to capture the lived experiences and personal perceptions rather than to measure motor difficulties objectively. Despite this limitation, the difficulties reported are consistent with those found in other studies^15 16 39 40^ supporting the validity of the participants’ accounts. However, to bridge this gap, future research should adopt a longitudinal design with objective reassessments, include a broad range of intrinsic and extrinsic factors and control for interventions in larger samples to help track motor difficulties over time. This approach could significantly enhance our understanding of the mechanisms behind persistent and remitted motor difficulties.

The flexibility of having face-to-face or phone interviews, potentially enhanced participation by overcoming practical barriers associated with in-person interviews. This flexibility strengthened the study by reaching and including many eligible participants. However, the use of different interview modes could impact the information shared.^46^ While phone interviews are typically shorter, they do not necessarily imply less information.^46^

### Conclusion

In this study, we explored the experiences of adults who faced motor difficulties in childhood and their transition into adulthood. Our findings suggest that individuals with motor difficulties find ways to navigate daily life without feeling limited. While some noted persistent difficulties, participants often attributed challenges to factors beyond motor skills. Parental support emerged as a crucial factor in their success and well-being, whereas a lack of school support highlights the need for targeted interventions to promote awareness and inclusive practices among teachers and schools.

Our study not only provides insights into the trajectory of motor difficulties but also contributes to a broader understanding of how individuals manage these challenges over time. Given the scarcity of research in this area, our findings offer valuable perspectives on the evolution of motor difficulties from childhood to adulthood. Despite the inherent limitations of this qualitative study, it comprehensively describes the experiences of growing up with motor difficulties.

By addressing contextual factors and fostering positive attitudes, we can empower individuals with motor difficulties to pursue their goals, enhancing their overall well-being and promoting full participation in society. Our study underscores the significance of social and environmental influences in shaping their lives, emphasising the importance of creating supportive environments and enhancing parental understanding to optimise outcomes for children with motor difficulties.

### Checklist reporting statement

The COREQ was used to ensure that essential information regarding the study was reported.^28^

## supplementary material

10.1136/bmjopen-2024-084346 online supplemental file 1

## Data Availability

Data are available on reasonable request.
