# Supplementary material for: Exploring adults’ recollections of growing up with childhood motor difficulties: a qualitative study using systematic text condensation
Source: BMJ Open. 2024 Aug 8;14(8):e084346. doi: 10.1136/bmjopen-2024-084346 (PMC11407206; doi:10.1136/bmjopen-2024-084346)
Supplement: online supplemental file 1 [file bmjopen-14-8-s001.pdf]

Supplementary file 1. Interview guide.

1. Tell me about the activities you like to do. Why do you like these activities? How often do you engage in these activities?
2. Tell me about the activities you wish you could or would like to do. What has stopped you from doing these activities?
3. When you were a child, you were assessed as "clumsy", or slow or fast when moving? Is this still so?

If answering that she/he still has motor difficulties, go to questions 4-7.
If answering that she/he no longer has motor difficulties, go to questions 8-10.
If answering that she/he has no own memory of having motor difficulties, go to questions 26-36.

If answering that she/he still has motor difficulties,

4. Is it any different now compared to when you were a child? Has this changed over time?
5. What activities do you find hard to do? Why?
   Self-care *(such as dressing, tying shoes, using utensils, shaving)*
   School and work activities *(such as handwriting/typing on the computer, planning the day, following recipes, gym class, crafts class)*
   Recreation/leisure activities *(sports, games, bowling, being a scout, driving a car, learning to drive a car/bike, playing tv/pc-games, swimming, playing a musical instrument, assembling IKEA furniture, renovating a home).*
6. Have you done the activity anyway? How have you solved it? Why did you persist with the activity?
7. Can you describe a typical day where your motor difficulties may impact your daily routine? You wake up in the morning until you go to bed at night.
   Probe for thoughts and feelings.
   ➔ Go to questions 11-25.

If answering that she/he no longer has motor difficulties, go to questions 8-10.

8. Were there any activities you found harder than others? Why?
9. Do you remember when it changed? Why? Have you gotten better or worse?
10. Is there anything you stopped doing because it became hard?

Continue here for still has and had motor difficulties as a child/youth.

11. Have you been able to participate in daily activities, leisure time activities and social activities as you wanted as a child and a youth?
    Today?
    If yes, is there anything specific that has made it possible? Why do you think that is?
    If not, what has stopped you from these activities? Why do you think that is?

12. *Describe a weekday when you were a child? Tell me about what you did with your family when you grew up.
    Did it change when you became a youth?

13. *Tell me about what you did on weekends/school breaks when you were a child
    And when you became a youth?
    Now?

14. *Tell me about the support you received as a child and youth from your family.
    From school?

Would you have liked more/less support? In another way? Tell me how

15. *What did you do during recess at school?
At ages 6-12 (primary and middle school)
At ages 13-15 (high school)
At ages 16-19 (upper secondary school)

16. Do you think your motor difficulties have affected what you do/socialise with your friends? If so, how?
Do you get comments about your difficulties?
Does it affect your participation in activities?

17. Do you think having motor difficulties affected how you feel about yourself? If you end up in challenging situations because of your motor difficulties, do you feel confident that you can solve the problem? If yes/now, why? What do you do when you end up in challenging situations? *Do you ask for help?*

18. Do you think that your motor difficulties have affected how others feel about you, now and/or when you were a child?
How?
If necessary, ask if he/she has been teased or bullied, if they have lots of friends, etc.

19. Do you think that your motor difficulties have influenced/affected your thoughts about being a parent? If so, how?
If you are a parent, how does your motor difficulties affect you as a parent?

20. *Tell me about the worst memory you have of a physical activity
Why this memory?
Feelings

21. *Tell me about the best memory you have of a physical activity
Why this memory?
Feelings

22. What is the hardest or most difficult thing about having motor difficulties?

23. If you were talking to a child who just found out that he or she has similar motor difficulties as you, what would you want him or her to know? What have you learned?

24. Anything else you would like to share about living with motor difficulties? Anything positive?

25. Is there anything else that you think we should study further?

If answering that they did not remember having motor difficulties:

If the participant did not remember having any motor difficulties, proceed to the next question

26. Have you been able to participate in daily activities, leisure time activities, and social activities as you have as a child and youth?
Today?
If yes, is there anything specific that has made it possible? Why do you think that is?
If no, what has stopped you from these activities? Why do you think that is?

If needed, or uncertainties, ask more specifically about, give examples:
Self-care *(such as dressing, tying shoes, using utensils, shaving);*
School and work activities *(such as handwriting/typing on the computer, planning the day, following recipes, gym class, crafts class);*
Recreation/leisure activities *(such as sports, games, bowling, being a scout, driving a car, learning to drive a car/bike, playing tv/pc-games, swimming, playing a musical instrument, assembling IKEA furniture, renovating a home).*
If needed, go back to questions about having/remembering motor difficulties

27. *Describe a weekday when you were a child? Tell me about what you did with your family when you grew up.
Did it change when you became a youth?
Tell me how a regular weekday is for you nowadays

28. *Tell me about what you did on weekends/school breaks when you were a child
And when you became a youth?
Now?

29. *Tell me about the support you received as a child and as a youth from your family.
From school?
Would you have liked more/less support? In another way? Tell me how

30. *What did you do during recess at school:
At ages 6-12 (primary and middle school)
At ages 13-15 (high school)
At ages 16-19 (upper secondary school)

31. Do you think that your motor difficulties as a child have affected how others feel about you? If so, how?

32. Do you get comments about how you move or how you perform motor tasks?
If necessary, ask if he/she has been teased or bullied, if they have lots of friends, etc.?

33. Do you think having had motor difficulties affected your feelings and confidence about yourself?
If yes/no, why?
What do you do if you find yourself in a challenging situation?
Do you ask for help?

34. How is your confidence in solving motor tasks? Tell me.

35. *Tell me about the worst memory you have of a physical activity
Why this memory?

Feelings

36. *Tell me about the best memory you have of a physical activity

Why this memory?

Feelings

37. Is there anything else that you think we should study further?
