# Supplementary material for: Exploring adults’ recollections of growing up with childhood motor difficulties: a qualitative study using systematic text condensation
Source: BMJ Open. 2024 Aug 8;14(8):e084346. doi: 10.1136/bmjopen-2024-084346 (PMC11407206; doi:10.1136/bmjopen-2024-084346)
Supplement: Supplementary file 2 [file bmjopen-2024-084346.reviewer_comments.pdf]

PEER REVIEW HISTORY

BMJ Open publishes all reviews undertaken for accepted manuscripts. Reviewers are asked to complete a checklist review form (**http://bmjopen.bmj.com/site/about/resources/checklist.pdf**) and are provided with free text boxes to elaborate on their assessment. These free text comments are reproduced below.

ARTICLE DETAILS

**TITLE (PROVISIONAL)**	Exploring adults' recollections of growing up with childhood motor difficulties – a qualitative study using Systematic Text Condensation
**AUTHORS**	Johansen, Kine; Zahlander, Johanna; Fäldt, Anna; Kirby, Amanda

VERSION 1 – REVIEW

**REVIEWER**	Lee, Daehyoung<br>University of Delaware College of Health Sciences, Health Behavior and Nutrition Sciences
**REVIEW RETURNED**	27-Jan-2024

**GENERAL COMMENTS**	Thank you for the opportunity to review this manuscript. I hope the authors find the following comments helpful to improve the overall quality of the manuscript.<br><br>ABSTRACT<br>Description of participants is unclear. Specify the inclusion criteria.<br><br>INTRODUCTION<br>Please address/answer the following.<br>What's the sustained impact of DCD on physical activity participation in adulthood?<br><br>Specific descriptions of successful & unsuccessful strategies in the literature should be addressed.<br><br>METHODS<br>Specify the classification approach by Hemgren and Persson. This is not a broadly accepted practice so some of the readers might have difficulty grasping this classification approach.<br><br>The research team excluded those with deficits in cognitive attention or social behaviors. These deficits are commonly referred to as co-occurring challenges in people w/ DCD. Please justify why and how the research team decided to establish this as an exclusionary criteria.<br><br>What was the quantifiable criteria that the research team used to determine the saturation of the interview data?<br><br>Specify what the Systematic Text Condensation is. Justify why this approach was relevant for the study.<br><br>Degree of individual variability among participants is high given the substantial time gap between the moments of diagnosis and study participation. It will be better if the authors described and compared different individual and contextual factors as a form of ecological assessment of the participants.

	RESULTS<br>Specify the results of motor ability assessments implemented in childhood. It's assumed that the severity of motor difficulties varied among participants. It might be interesting to see how it relates to gestational age and current motor abilities in adulthood.<br><br>The two overarching themes generated are too broad. I guess the fundamental problem lies in the establishment of a broad research question. The research team should narrow down the research topic that addresses a specific outcome variable.<br><br>There are not enough quotes to support each category. The research team should thoroughly review the interview data and provide more in-depth analyses on each category. There probably exists confirmation bias if the themes were dominantly generated by predetermined idea.<br><br><br>DISCUSSION<br>Authors briefly discussed the differentiation between those who do have sustained motor difficulties and who do not. There should be more discussion as to why and how the childhood motor difficulties may or may not persist into adulthood. What individual and environmental factors play a role in creating this gap?<br>Parental support and gender difference were discussed but there exists a lot more impactful factors they should explore, such as peer interaction, influence of siblings, co-occurring conditions (sensory overload, anxiety, attention deficit), school/home education, learning opportunities, etc.<br><br>Motor difficulties accompanied by DCD may disappear before adulthood. The authors mentioned the absence of reassessment as a limitation. It needs to be further discussed.<br><br>Overall, it's difficult pinpoint the topic of this study. What's the outcome variable(s) that this study investigated? Is it coping strategies for physical activity participation or general quality of life? Authors should narrow down the focal point and discuss the study topic in depth.

**REVIEWER**	Samuel, Reema<br>Christian Medical College and Hospital Vellore, Psychiatry
**REVIEW RETURNED**	22-Feb-2024

**GENERAL COMMENTS**	Exploring how adults with childhood motor difficulties recall their upbringing – a qualitative study: bmjopen-2024-084346<br>Reviewer Comments<br><br>Thank you for the opportunity to review your article. The authors have explored the experience of living with a motor difficulty and its impact on daily life. The findings of the study are easily relatable for professionals working in the field of rehabilitation. I would like to provide the following comments.<br><br>1. The manuscript is well- written. The methodology, data analysis and results section contain all pertinent information. In keeping with the Systematic Text Condensation strategy, the progression from initial themes and codes to the final synthesis in the discussion section is clear.<br><br>2. Kindly consider including the analytical method in the title: e.g. Exploring how adults with childhood motor difficulties recall their

	upbringing – a qualitative study using Systematic Text Condensation.<br><br>3. I feel Table 2 could be expanded to include condensates and quotations from each code group. This would enable readers who are unfamiliar with the methodology, to get a concise understanding of the results.<br><br>4. The interview guide includes questions related to impact of motor difficulties in parenting. This has not been mentioned in the results. As this would be an important life area for participants, consider adding the related themes to the results section. Also, any other relevant, but minor themes could be briefly mentioned.<br><br>All the best!

VERSION 1 – AUTHOR RESPONSE

Revision 1	Comments to author	Authors reply	Changes page – line
Reviewer 1	**Abstract**		
	Description of participants is unclear. Specify the inclusion criteria.	The description of participants has been updated.	Page 2, line 31-34
	**Introduction**		
	What's the sustained impact of DCD on physical activity participation in adulthood?	We have added a sentence about DCD and physical activity in adulthood in the introduction and provided an example on how participation physical activities may be affected.	Page 5, line 100–102
	Specific descriptions of successful& unsuccessful strategies in the literature should be addressed.	We have added this in the introduction.	Page 5, line 110-113

	**Methods**		
	Specify the classification approach by Hemgren and Persson. This is not a broadly accepted practice so some of the readers might have difficulty grasping this classification approach.	We have described how the categories Hemgren and Perssonused in their study were defined.	Page 6, line 135–144
	The research team excluded those with deficits in cognitive attention or social behaviors. These deficits are commonly referred to as co-occurring challenges in people w/ DCD. Please justify why and how the research team decided to establish this as an exclusion criteria.	We agree with the reviewer that co-occurrence is the rule rather than the exception, but we wantedto learn more about how motor difficulties affected the individual. Therefore, to minimize the possibility that the experiences was caused by other factors we chose to include only those who had only motor problems at 6.5 years of age. We have added this to the text.	Page 7, line 150-153
	What was the quantifiable criteria that the research team used to determine the saturation of the interview data?	We did not have a quantifiable criterion, but conducted interviews until we had sufficient empirical data to answer our aim and until the new data did not add more information compared to the previous data, i.e. until saturation was achieved.<br><br>The concept of saturation is to secure that analysis are drawn from sufficiently rich and heterogeneous empirical data (Malterud, 2012). We did not aim to provide a complete description of all possible experiences of motor difficulties, but rather to achieve an information-rich sample containing different experiences of the specific situation we wanted to explore. According to Malterud (2012), ‘a rule of thumb is that the sample should be sufficiently large and varied to elucidate our aim’.<br><br>Saturation can also be understood as information power (Malterud et al., 2016). Information power indicates that the more information the sample holds, the lower number of participants is needed. Hence, information poweris also dependant on the quality of	Page 8, line 183–186

		the dialogue (Malterud et al., 2016).<br><br>In our study, the interviews were conducted by the first author, who is knowledgeable about motor difficulties and has extensive experience in meeting and talking to patients and parents of children with motor difficulties. After conducting 14 interviews we experienced that our data contained varied descriptions of the participants experiences and that the last interviews did not contribute with new knowledge in regards to elucidating our aim. Hence, saturation was reached. We have added this to the manuscript.	
	Specify what the Systematic Text Condensation is. Justify why this approach was relevant for the study.	We have added a sentence about what the systematic text condensation (STC) is and why it was suitable for the study. Additionally, we have added a table (Table 1) explaining the analysis procedure.	Page 8, line 189-191<br><br>Page 9, Table 1
	Degree of individual variability among participants is high given the substantial time gap between the moments of diagnosis and study participation. It will be betterif the authors described and compared different individual and contextual factors as a form of ecological assessment of the participants.	The reviewer raises an important point regarding the high variability among participants, which can be attributed to the significant time gaps between when their motor difficulties were identified and participation in our study. We recognize the value of conducting a detailed ecological assessment to explore individual and contextual factors as suggested. However, our study was not designed to collect this specific type of data, and consequently, we do not possess the data needed to perform such an analysis. We acknowledge that development is indeed influenced by a variety of factors and agree that tracking these over time using an ecological approach would provide a more comprehensive picture, significantly enriching our understanding. While our current study design does not include this type of data collection, we	

		recognize its importance and strongly recommend that future research in this field incorporates ecological assessments to address these complexities. This approach would undoubtedly contribute valuable insights into the variability and dynamics of development over time.	
	**Results**		
	Specify the results of motor ability assessments implemented in childhood. It's assumed that the severity of motor difficulties varied among participants. It might be interesting to see how it relates to gestational age and current motor abilities in adulthood.	We have revised the table on sample characteristics (Table 2) to present results at the group level, enabling a clearer comparison between participants with or without persistent motor difficulties with respect to variablessuch as gestational age.<br>Additionally, we have included a sentence in the manuscript describing the characteristics of participants who have persistent motor problems.	Page 10, line 228-230<br><br>Page 10, Table 2
	The two overarching themes generated are too broad. I guess the fundamental problem lies in the establishment of a broad research question. The research team should narrow down the research topic that addresses a specific outcome variable.	Thank you for this important feedback. In response, we have revised our analysis and narrowed the themes to more accurately reflect the results, focusing specifically on the progression from childhood to adulthood. This refinement has allowed us to develop new theme names and category titles that are both more precise and indicative of the longitudinal scope of our study. We believe these adjustments significantly enhance the clarity and applicability of our findings.	Page 11-16, line 234-398
	There are not enough quotes to support each category. The research team should thoroughly review the interview data and provide more in-depth analyses on each category. There probably exists confirmation bias if the themes were dominantly generated by predetermined idea.	We appreciate your concerns regarding the need for sufficient evidence to support each category and the potential for confirmation bias in our thematic analysis.<br><br>To mitigate these risks, our analysis strictly adhered to the pragmatic procedures outlined by the STC method (Malterud, 2012). Initially, we immersed ourselves in the data to gain a comprehensive understanding (Total impression).	Page 12–16, line 255–373<br><br>Page 9, Table 1

		Subsequently, we identified meaning units and sorted them into code groups (Identifying and sorting meaning units). These units were then condensed into concise descriptions that integrate all perspectives, narrated as if by a single participant (Condensation). Finally, we synthesized these descriptions to develop overarching interpretations and insights (Synthesizing and summarizing).<br><br>A key strength of STC is evident in its condensation step—where empirical data are reduced to a decontextualized selection of meaning units sorted into thematic code groups across individual participants. These units are then transformed into a more abstract format. During this process, we use a first-person narrative to ensure that each participant who provided information on a specific issue is represented. This methodological rigor ensures a comprehensive representation and mitigates the risk of bias, thereby enhancing the nuance and thoroughness of our analysis.<br><br>In response to your specific feedback, we have added more quotes across the categories to better substantiate our findings. Additionally, we have included a new table (Table 1), which provides a detailed example of the STC process. These enhancements aim to improve the transparency and credibility of our study, ensuring that each category is well-supported and accurately reflects the range of data collected.	
	**Discussion**		
	Authors briefly discussed the differentiation between those who do have sustained motor difficulties and who do not. There	We have expanded our discussion to include additional factors influencing motor development, addressing its complex nature. We	Page 19, line 474-490

	should be more discussion as to why and how the childhood motor difficulties may or may not persist into adulthood. What individual and environmental factors play a role in creating this gap?<br><br>Parental support and gender difference were discussed but there exists a lot more impactful factors they should explore, such as peer interaction, influence of siblings, co-occurring conditions (sensory overload, anxiety, attention deficit), school/home education, learning opportunities, etc.	hope this addition adequately addresses the gaps identified in our initial manuscript.	
	Motor difficulties accompanied by DCD may disappear before adulthood. The authors mentioned the absence of reassessment as a limitation. It needs to be further discussed.	We have expanded the discussion on the limitations related to the lack of objective reassessment of motor difficulties in adulthood andits implications for confirming DCD diagnoses to address your comments. We also emphasize the importance of future longitudinal studies that incorporate such reassessments to better understand the progression or resolution of these difficulties.	Page 20, line 516-530
	Overall, it's difficult pinpoint the topic of this study. What's the outcome variable(s) that this study investigated? Is it coping strategies for physical activity participation or general quality of life? Authors should narrow down the focal point and discuss the study topic in depth.	Thank you for your valuable feedback. We have carefully revised our analysis and updated the results to more accurately align with the study's aim. These changes aim to clarify the focus of our research, enhancing the presentation and understanding of our findings.	Page 11-16, line 234-398
**Reviewer 2**	The manuscript is well-written. The methodology, data analysis and results section contain all pertinent information. In keeping with the Systematic Text Condensation strategy, the progression from initial themes and codes to the final synthesis in the discussion section is clear.	We thank the reviewer for reading and revising our manuscript.	

	Kindly consider including the analytical method in the title: e.g. Exploring how adults with childhood motor difficulties recall their upbringing – a qualitative study using Systematic Text Condensation.	We have added "using Systematic Text Condensation" in the title as suggested by the reviewer.	Page 1, line 3-4
	I feel Table 2 could be expanded to include condensates and quotations from each code group. This would enable readers who are unfamiliar with the methodology, to get a concise understanding of the results.	Thank you for your suggestion. We have added a new table (Table 1) to illustrate the analysis procedure according to STC, hoping to aid those unfamiliar with the method.	Page 9, Table 1
	The interview guide includes questions related to impact of motor difficulties in parenting. Thishas not been mentioned in the results. As this would be an important life area for participants,consider adding the related themesto the results section. Also, any other relevant, but minor themes could be briefly mentioned.	Thank you for highlighting the importance of addressing the impact of motor difficulties on parenting within our study. We have updated the results section to include a specific mention of this issue. Additionally, we have thoroughly reviewed our data to ensure that all relevant themes, including minor ones, are adequately represented. This ensures that no significant topics discussed by participants have been omitted from our report.	Page 12, line 267-268

References

Malterud, K. (2012). Systematic text condensation: A strategy for qualitative analysis. *Scandinavian Journalof Public Health*, *40*(8), 795–805. https://doi.org/10.1177/1403494812465030

Malterud, K., Siersma, V. D., & Guassora, A. D. (2016). Sample Size in Qualitative Interview Studies: Guidedby Information Power. *Qualitative Health Research*, *26*(13), 1753–1760. https://doi.org/10.1177/1049732315617444

VERSION 2 – REVIEW

**REVIEWER**	Samuel, Reema<br>Christian Medical College and Hospital Vellore, Psychiatry
**REVIEW RETURNED**	14-May-2024

**GENERAL COMMENTS**	I would like to apologise for the inadvertent delay in providing my comments. The authors have satisfactorily addressed the earlier suggestions. I do have a few more suggestions.

1. There is better clarity of the study question in the objectives section of the abstract as well as at the end of introduction. Consequentially, the current study **title** seems too broad- ‘how adults with childhood motor difficulties *recall their upbringing*’. Kindly consider if the title can be more specific and reflective of your study question.
2. I agree with both Reviewer 1 and the authors on the importance of ecological assessments to understand the trajectory of motor difficulties over time. Perhaps this can also be added as a specific recommendation at the end of your discussion section (line 526- 530).
3. The manuscript is a bit over the word limit. I can understand it is very difficult to balance between the brevity of writing and the quality of information in the manuscript, especially for qualitative studies. A few suggestions I have is to cut down on repetitive quotes in the **results** section, e.g. line 372-373 and other themes under ‘coping strategies’ is already described well in Table 1. Also, where illustrative quotes are provided, similar descriptions in the text about the content of the quotes can be reduced. E.g. line 313-319. Another idea is to add a detailed table of illustrative quotes and themes to the supplementary file so that interested readers have access to information, while the readability of the main manuscript is retained.
4. I feel the **discussion** section can be modified to allow for more cohesive flow of content. E.g.

   -Lines 400-408 and lines 490-504 are repetitions of similar concepts.
   -Lines 448-450 and lines 470-473, are paragraphs which end with recommendations/ implications for practice. The earlier paragraphs do not end with recommendations.
   -Lines 474-482 conceptually ties in with the author recommendations given in lines 506-515.
   The authors can consider restructuring the discussion section as

   - Statement of major finding (this is done well, but

	repeats in various places)<br><br>• Theme-wise discussion of findings, with explanations for contrary/ new findings (whether implications of findings need to be written up according to themes or as an overall synthesis towards the end of discussion is upto the authors, but kindly follow a consistent pattern.<br><br>5. The **limitation** section has an elaborated section on ethical implications- 536-547. I think this portion is better suited under the methodology section, if at all it needs to be added. I understand why the authors feel the need to elaborate this. But as a reader, I personally feel that if the study has been approved by the Institutional Ethics Board and if informed consent has been obtained from the participants, I would be satisfied that no breach of ethical practice guidelines has happened. Hence, this section can be shortened and added earlier in the manuscript.<br><br>I thank Reviewer 1, whose suggestions helped me conceptualise this study in a broader context. I look forward to the revised manuscript.

VERSION 2 – AUTHOR RESPONSE

Below youwillfindourresponsestothecommentspointbypoint.Allchanges havebeenhighlighted using the track change function, and the reference list has been updated accordingly.

1. The current study title seems too broad- ‘how adultswith childhood motor difficulties recall theirupbringing’.Kindlyconsiderifthetitlecanbemorespecificandreflectiveofyourstudy question.

   Response: We agree that the title wastoo broad anddid not accurately reflect our study's findings.Therefore,wehaverevisedthetitleto:"Exploringadults’recollectionsofgrowing up with childhood motor difficulties – a qualitative study using Systematic Text Condensation”

2. IagreewithbothReviewer1 andtheauthorsontheimportanceofecologicalassessmentsto understand the trajectory of motordifficultiesover time. Perhapsthis canalso be added as a specific recommendation at the end of your discussion section (line 526- 530).

Response: Thankyouforthiscomment.Wehaveaddedtherequestedrecommendation.The revised sentence now states: “Looking ahead, a shift from impairment-based approaches to strength-based ecological or environmental assessments and interventions is recommended, aiming to enhance social and community participation for individuals with DCD.[16]”

3. The manuscript is a bit over the word limit. I can understand it is very difficult to balance betweenthebrevityofwritingandthequalityofinformationinthemanuscript,especially for qualitative studies.

   Response: Thank you for this vital comment. We have considered the different options you presented. To increase readability without losing transparency and the reflection of participants'voices,wehaveremovedredundantinformationthatwaspresentedbothinthe text and in the quotes. We have retained quotes that describe something different orprovide additional insights compared to the main text. Furthermore, we have kept quotes used in both the table and the text to clearly demonstrate the analysis process and maintain a clear link to the results.
4. Ifeelthediscussionsectioncanbemodifiedtoallowformorecohesiveflowof content.

   Response:Thankyouforthesuggestion.Wehaverearrangedthediscussionsectionto enhance cohesiveness, organizing it theme-wise to better discuss the findings.

5. The limitation section has an elaborated section onethical implications-536-547. I think this portion is better suited under the methodology section, if at all it needs to be added. I understandwhytheauthorsfeeltheneedtoelaboratethis.Butasareader,Ipersonallyfeel thatifthestudyhasbeenapprovedbytheInstitutionalEthicsBoardandifinformedconsent has been obtained from the participants, I would be satisfied that no breach of ethical practice guidelines has happened. Hence, this sectioncan be shortened and added earlier in the manuscript.

   Response: We agree that the description of the ethical implications of interviewing participants unaware of their prior involvement is redundant as a limitation. We have shortenedthissectionandrelocatedittotheData collectionsectionofthemethodology.
